# Mapping the moral boundaries of biological engineering

**DOI:** 10.1186/1754-1611-3-7

**Published:** 2009-05-08

**Authors:** Zachary N Russ

**Affiliations:** 1 Fischell Department of Bioengineering, Room 2330 Jeong H. Kim Engineering Building (Bldg. #255), University of Maryland, College Park, MD 20742, USA

## Abstract

The following essay was written by a sophomore undergraduate student majoring in Bioengineering at the University of Maryland, Mr. Zachary Russ. Mr. Russ was one of 174 students who submitted a 1000–1200 word essay to the 4th Annual Bioethics Contest sponsored by the Institute of Biological Engineering (IBE). A group of professionals in Biological Engineering assessed and ranked the essays in a blinded process. Five semi-finalists were invited to present their essays at a session at the annual meeting of IBE in Santa Clara, CA on March 21, 2009. Five judges scored all the presentation at the annual meeting and selected Mr. Russ's contribution as the overall winner (1st Place).

## Essay

"It worked" was the understated comment of Manhattan Project scientific director J. Robert Oppenheimer after witnessing the first nuclear detonation in 1945. Only later did Oppenheimer reveal that in his mind he had experienced a far grimmer reaction, recalling a passage from the *Bhagavad Gita*: "Now I am become Death, the destroyer of worlds." Oppenheimer's remorse at a moment of scientific triumph was hardly unique. In 1939, after conducting an early experiment at Columbia University that confirmed scientists' ability to split atoms, the physicist Leo Szilard observed, "We turned the switch, saw the flashes, then switched everything off and went home. That night I knew the world was headed for sorrow." In 1969, Erwin Chargaff, Professor of Biological Chemistry at Columbia, noted "We manipulate nature as if we were stuffing an Alsatian goose. We create new forms of energy; we make new elements; we kill crops; we wash brains. I can hear them in the dark sharpening their lasers."[1]

The pursuit of knowledge is as old as humankind itself. Our innate curiosity is reflected in myriad scientific advances: higher standards of living, longer life spans, eradication of diseases, exploration of the universe, and more. Yet we must never forget the horrors that sometimes accompany technological breakthroughs. In 1984, a pesticide plant in Bhopal, India – established to aid local agriculture – accidentally released extremely toxic chemicals, killing 3,800 and injuring thousands more [2]. Similarly, the 1986 Chernobyl nuclear reactor disaster killed dozens and irradiated thousands, forced the evacuation and resettlement of more than 330,000 people, and turned 30 kilometers around the plant into a radioactive no-man's-land [3]. Even a small experiment in 1957 in a research lab in Brazil to create a more productive honey bee population went famously awry as the accidental release of 26 Tanzanian queen "killer" bees led to widespread ecological damage in the Americas and the loss of dozens of human lives [4].

All these incidents and many others stemmed from a basic failure to fully evaluate the implications of technological changes. In modern jargon, they represent "failures in safety protocols" and are a stark reminder that as we pursue scientific knowledge, we must be prepared for whatever accidents or contingencies may result. Consequently, I strongly believe the discipline of biological engineering needs its own code of ethics. Medicine's ancient code of ethics, the Hippocratic Oath, offers this admonition to doctors – "First, do no harm." It is a powerful reminder to all that whatever we do to help humanity can have unexpected and undesirable side effects. Therefore, we bioengineers also have a responsibility to our fellow human beings and to our environment to develop and live by a code of ethics.

What form should our code take? Biological engineering, a blossoming field at the crossroads of so many disciplines – biology, ecology, chemistry, physics, medicine, and engineering – exposes itself to unprecedented moral challenges by making use of so many techniques, including genetic engineering, clinical research, and animal experimentation. No other field encompasses so many potential pitfalls. It is for this reason that biological engineering needs its own unique code of ethics – it cannot simply derive its ethics from a parent field, because the moral questions posed to bioengineers are not a subset of any other group. Medicine generally does not deal with the possibility of environmental impact [5](although, ironically, that assertion now may be changing as prescription medications end up in our waterways[6]); biology generally avoids patient research; and chemists and physicists generally need worry only about laboratory accidents and environmental contamination. Yet we biological engineers will have to routinely step over established boundaries into uncharted moral territory in carrying out assignments. It would be useful for us to have a map by which to align our moral compasses.

So what ethical lines should be drawn on this map? A bioengineer is, first and foremost, a human being. As such, the first element of the code of ethics should include the basic reminder that in carrying out his or her work, the bioengineer assumes an enormous responsibility for the safety and well-being of other people. Designs must incorporate safeguards, cautionary procedures, and contingency plans for the possibility of catastrophic failure. A pioneering example was the first Apollo space mission, which mandated a three-week quarantine for the returning astronauts on the chance that they might return carrying some unforeseen biological agent from the surface of the moon [7]. Similarly, a bioengineering oversight could result in the release of invasive species or infectious agents, a critical medical device failing to sustain patients, or an imaging device causing misdiagnoses. These are the most serious mistakes a bioengineer can make. Therefore, when it comes to unintended consequences to the public and the environment, attention to detail is of paramount importance.

The consideration of safety is found mainly in engineering codes [8-10]. Related considerations about calling attention to misconduct and staying within the bounds of one's competence would also make sense, as a subset of the previous rule.

However, the bioengineer is likely a researcher as well as an engineer, as one can see from the numerous research papers contained in the *Journal of Biological Engineering*. Scientific ethical systems focus on honesty – not just honesty about one's qualifications or objective presentation of facts, but also the explicit prohibition against fabrication, plagiarism, and exploitation of others [11]. The need for these rules is obvious, as illustrated by several recent high-profile research scandals, such as the embryonic stem-cell cloning scandal [12]. Careers are destroyed and reputations of entire fields are tarnished every time one of these cases emerges.

Besides the basics, bioengineers may also find themselves in more complex moral situations such as live vertebrate testing, which carries a whole set of ethical concerns that are currently addressed by publicly available guidelines [13]. Clinical research in the U.S. is overseen by the Department of Health and Human Services and the Food and Drug Administration, where consideration of human welfare is always critically important [14,15]. However, because many clinical trials are done overseas and sometimes with minimal regulation, it is essential that our code of ethics ensure that all patients are well-cared for and not worse off than if they had not entered the study. Currently, in the U.S., genetic engineering is mostly unregulated, so special care needs to be taken in designing guidelines to handle the human species with appropriate care and respect. Although human genetic enhancements are a long way away, it is preferable to begin discussing and examining the subject now, setting down what is and is not acceptable change in the human genetic composition, rather than waiting until after the first genetic enhancement has been made.

Finally, with the explosive growth of bioengineering as a separate college major and professional discipline, there will no longer be as many bioengineers who have been taught ethical considerations derived from other fields. It will be our responsibility to teach them. Since we must provide these neophytes a code of ethics, why not teach one that accurately reflects future needs as varied as the field itself? For a discipline with as much power to change the world as nuclear physics before it, bioengineering ethicists certainly will have their work cut out for them. We must remember that with great power comes great responsibility.

## Authors' contributions

ZR wrote the essay and read and approved the overall manuscript.
